# Molecular co-evolution of a protease and its substrate elucidated by analysis of the activity of predicted ancestral hatching enzyme

**DOI:** 10.1186/1471-2148-13-231

**Published:** 2013-10-25

**Authors:** Mari Kawaguchi, Koji Inoue, Ichiro Iuchi, Mutsumi Nishida, Shigeki Yasumasu

**Affiliations:** 1 Atmosphere and Ocean Research Institute, The University of Tokyo, 5-1-5 Kashiwanoha, Kashiwa, Chiba 277-8564, Japan; 2 Research Fellow of the Japan Society for the Promotion of Science (JSPS), Chiyoda-ku, Japan; 3 Department of Materials and Life Sciences, Faculty of Science and Technology, Sophia University, 7-1 Kioi-cho, Chiyoda-ku, Tokyo 102-8554, Japan; 4 Current address: Department of Materials and Life Sciences, Faculty of Science and Technology, Sophia University, 7-1 Kioi-cho, Chiyoda-ku, Tokyo 102-8554, Japan

**Keywords:** Co-evolution, Hatching enzyme, Astacin family, Egg envelope, Chorion, ZP domain, Ancestral hatching enzyme

## Abstract

**Background:**

Hatching enzyme is a protease that digests the egg envelope, enabling hatching of the embryo. We have comprehensively studied the molecular mechanisms of the enzyme action to its substrate egg envelope, and determined the gene/protein structure and phylogenetic relationships. Because the hatching enzyme must have evolved while maintaining its ability to digest the egg envelope, the hatching enzyme-egg envelope protein pair is a good model for studying molecular co-evolution of a protease and its substrate.

**Results:**

Hatching enzymes from medaka (*Oryzias latipes*) and killifish (*Fundulus heteroclitus*) showed species-specific egg envelope digestion. We found that by introducing four medaka-type residue amino acid substitutions into recombinant killifish hatching enzyme, the mutant killifish hatching enzyme could digest medaka egg envelope. Further, we studied the participation of the cleavage site of the substrate in the species-specificity of hatching enzyme. A P2-site single amino acid substitution was responsible for the species-specificity. Estimation of the activity of the predicted ancestral enzymes towards various types of cleavage sites along with prediction of the evolutionary timing of substitutions allowed prediction of a possible evolutionary pathway, as follows: ancestral hatching enzyme, which had relatively strict substrate specificity, developed broader specificity as a result of four amino acid substitutions in the active site cleft of the enzyme. Subsequently, a single substitution occurred within the cleavage site of the substrate, and the recent feature of species-specificity was established in the hatching enzyme-egg envelope system.

**Conclusions:**

The present study clearly provides an ideal model for protease-substrate co-evolution. The evolutionary process giving rise to species-specific egg envelope digestion of hatching enzyme was initiated by amino acid substitutions in the enzyme, resulting in altered substrate specificity, which later allowed an amino acid substitution in the substrate.

## Background

Many proteins co-evolve as a result of protein-protein interaction, and maintain or diversify their functions. This interaction is often complicated, so elucidation of the co-evolutionary process is difficult in many cases. The hatching enzyme of teleost species is secreted by the embryo to digest the egg envelope that surrounds the embryo and protects it from environmental, chemical, and mechanical stresses [1-4]. Considering that hatching enzyme would have evolved while maintaining the ability to digest the egg envelope, the hatching enzyme-egg envelope protein pair is a good model system for understanding molecular co-evolution of a protease and its substrate.

Both egg envelope protein and hatching enzyme have been studied in the model fish medaka (*Oryzias latipes*). The egg envelope consists of thick inner layers and a thin outer layer. The inner layers are composed of two groups of subunit proteins called ZI-1,2 and ZI-3 [5], which are classified into vertebrate-common egg envelope protein groups ZPB and ZPC, respectively [6,7]. ZI-1,2 are heterogeneous glycoproteins derived from the precursors choriogenin H and H minor. ZI-3 is a homogenous glycoprotein derived from the precursor choriogenin L [8-10]. Both ZI-1,2 and ZI-3 have a zona pellucida (ZP) domain consisting of about 260 amino acids, with eight conserved cysteine residues [11]. Additional characteristics of ZI-1,2 include a Pro-Xaa-Yaa repeat region and a trefoil domain upstream of the ZP domain.

Hatching enzyme is a member of the astacin metallo-protease family. Medaka possesses two types of hatching enzymes, high choriolytic enzyme (HCE; choriolysin H; EC 3.4.24.67) and low choriolytic enzyme (LCE; choriolysin L; EC 3.4.24.66) [12-14]. At hatching, both enzymes simultaneously act on the egg envelope and completely solubilize its inner layers [2]. The thin filamentous outer layer remains undigested and is broken by embryo movement. Studies have shown that the molecular mechanism of the enzymes is as follows: HCE swells the envelope by cleaving the N-terminal region of egg envelope proteins, especially the Pro-Xaa-Yaa repeat region on ZI-1,2, into small pieces [15,16]. LCE solubilizes the HCE-swollen envelope by cleaving only two sites, the N-terminal of the ZP domain of ZI-3, and the middle of the ZP domain of ZI-1,2 [16]. Because ZP domains assemble each other to form a higher-order architecture as the egg envelope [17,18], cleavage of the middle of the ZP domain by LCE may change the higher-order structure of the egg envelope and lead to its complete digestion [16]. Indeed, our group recently uncovered the importance of the middle region of the ZP domain (mid-ZPd) for solubilization, as demonstrated by the following findings [19]: the nine-spined stickleback possessed two types of LCE, LCEα and LCEβ, formed by gene duplication. LCEα cleaved the N-ZPd site but not the mid-ZPd site, while LCEβ cleaved only the mid-ZPd site but not the N-ZPd site. When only the N-ZPd site was cleaved by LCEα, the egg envelope was not solubilized, but remained swollen. The swollen egg envelope was solubilized only after the mid-ZPd site was cleaved by LCEβ. Thus, the mid-ZPd region is a key site for egg envelope solubilization.

Comparative studies have shown that the egg envelope digestion mechanism is conserved between medaka and *Fundulus* species. *Fundulus* possesses two isoforms of HCE, called FHCE1 and FHCE2, both of which swell the egg envelope [20]. *Fundulus* LCE then solubilizes the swollen envelope completely [20,21]. The positions of cleavage sites for *Fundulus* HCE (FHCE1/2) and LCE (FLCE) are similar to those of medaka HCE (MHCE) and LCE (MLCE) [21].

Recently, we identified both cross-species similarities and differences in the modes of action of hatching enzymes from medaka and *Fundulus*. When incubated with *Fundulus* hatching liquid containing both HCEs and LCE, the medaka egg envelope was swollen, but not solubilized [20]. In addition, we found that isolated *Fundulus* HCEs swelled the medaka egg envelope, but that isolated *Fundulus* LCE did not digest the swollen medaka egg envelope. Therefore, mode of action of HCE is common to both *Fundulus* and medaka, but the action of LCE in egg envelope digestion is substrate-specific.

In the present study, we identified the amino acid residues of LCE responsible for conferring the specificity in egg envelope digestion, and discuss a molecular co-evolutionary pathway for hatching enzyme and egg envelope protein in a teleostean lineage, based on data obtained from the reconstruction and activities of probable ancestral forms of teleostean LCEs.

## Results

### Species specificity of hatching enzyme from medaka and *Fundulus*

**Figure 1:**
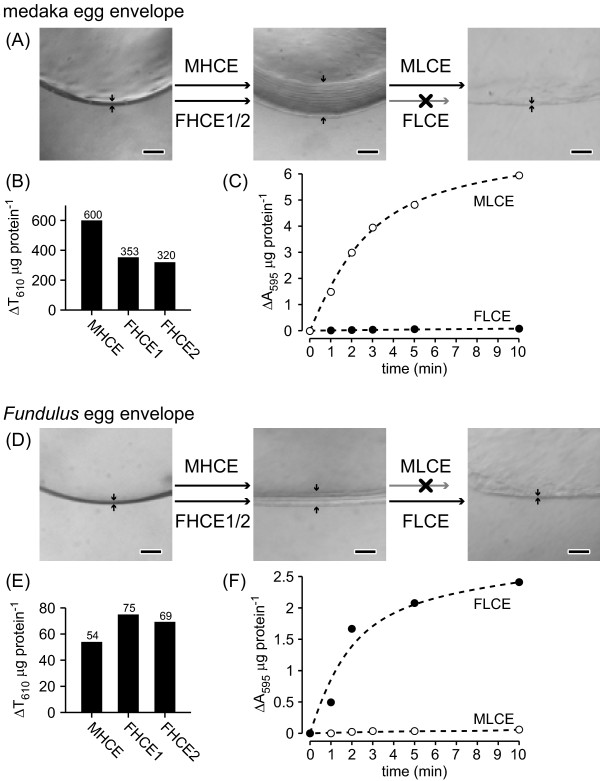
**Cross-species digestion of egg envelope by hatching enzymes. (A)** Photographs of sections of intact fertilized medaka egg envelope, and those treated with hatching enzymes, HCEs, and LCEs. Arrows indicate the thickness of egg envelope. The medaka egg envelope was swollen either by purified medaka HCE (MHCE) or by purified *Fundulus* HCE (FHCE1/2). The swollen envelope was solubilized by purified MLCE, but not by purified FLCE. **(B)** Swelling activity of HCEs towards the medaka egg envelope, and **(C)** time course of the solubilization of the swollen envelope by LCEs. **(D)** Photographs of sections of intact fertilized egg envelope from *Fundulus* and those treated with hatching enzymes, HCEs, and LCEs. Arrows indicate the thickness of the envelope. The *Fundulus* envelope was also swollen either by MHCE or by FHCE1/2. The swollen envelope was solubilized by FLCE, but not by MLCE. Swelling activity of HCEs towards *Fundulus* envelope **(E)**, and time course of the solubilization of the swollen envelope by LCEs **(F)**. Scale bars, 100 μm.

As shown in Figures 1A and 1D, HCE from medaka *O. latipes* (MHCE) and *Fundulus heteroclitus* (FHCE1 and FHCE2) swelled the egg envelope of both species. Activities of HCE in the cross-species or xenogenic combinations were somewhat lower (Figure 1B and 1E), but were sufficient to swell the egg envelope. Thus, the action of HCE showed low substrate specificity in both medaka and *Fundulus*.

In contrast to HCE, LCE could only digest the swollen envelope of the species from which it originated (Figure 1A and 1D). Medaka LCE (MLCE) sharply increased solubilization of the swollen medaka egg envelope with incubation time, whereas MLCE only slightly solubilized that of *Fundulus* (Figure 1C). Similarly, *Fundulus* LCE (FLCE) efficiently solubilized the swollen envelope of *Fundulus*, but not that of medaka (Figure 1 F). These results suggested that LCE activity is substrate-specific in medaka and *Fundulus*.

### Identification of key amino acid residues responsible for the species-specificity of LCE activity

#### (i) Egg envelope protein

**Figure 2:**
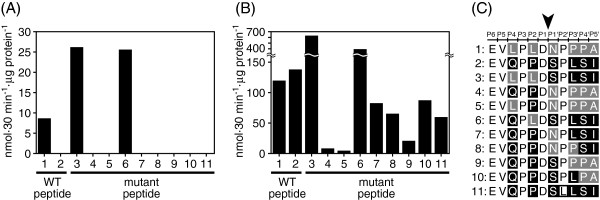
**Identification of important residues in egg envelope protein for species-specific egg envelope digestion.** Cleavage activity of purified FLCE **(A)** and MLCE **(B)** towards wild-type cleavage site peptides, mid-ZPd, of *Fundulus* and medaka egg envelope proteins (peptides 1 and 2), and towards their mutant peptides (peptides 3–11). Specific activities are expressed as nmol · 30 min^-1^ · μg protein^-1^. The sequences are listed in **(C)**. The amino acid residues mutated to *Fundulus*-type and medaka-type are shown with gray and black, respectively. The cleavage site is shown with an arrowhead, and the amino acid positions from the cleavage site are indicated at the top of the sequence according to the nomenclature for the interaction of proteases with their substrates [22].

Species-specific egg envelope digestion in *Fundulus* or medaka would be established by amino acid changes both in egg envelope protein and in hatching enzyme LCE. We first focused on the key LCE cleavage sites in the envelope protein, the mid-ZPd region. We identified six substitutions in 11 amino acid residues of the mid-ZPd regions of both *Fundulus* and medaka, and synthetic peptides were then designed from the sequences around the mid-ZPd cleavage site (Figure 2C, peptides 1 and 2).

FLCE cleaved the *Fundulus* mid-ZPd peptide (peptide 1), but not the medaka peptide (peptide 2) (Figure 2A), confirming the species-specificity of egg envelope digestion. MLCE digested the mid-ZPd peptides from both *Fundulus* and medaka (Figure 2B), suggesting that the species specificity of MLCE does not depend on the peptide sequence around the cleavage site. Because egg envelope has an insoluble macro-molecular structure, the species specificity of MLCE for cleavage of the intact swollen egg envelope may be due to the enzyme-to-substrate interaction in a higher-ordered conformation.

Next, we constructed mutant peptides of the mid-ZPd region whose residue(s) were substituted with either *Fundulus*- ormedaka-type residues (peptides 3–10). As shown in Figure 2A, FLCE only cleaved peptides 1, 3, and 6, all of which possess the common residue L at the P2 site [22]. L to P substitution at the P2 site of the *Fundulus* peptide significantly reduced the activity of FLCE. In contrast, P to L substitution at the corresponding site of the medaka peptide enabled FLCE activity. These results suggested that the FLCE-specific digestion could be explained by the presence of L at the P2 site in the FLCE-cleavage site on the substrate.

#### (ii) Hatching enzyme

**Figure 3:**
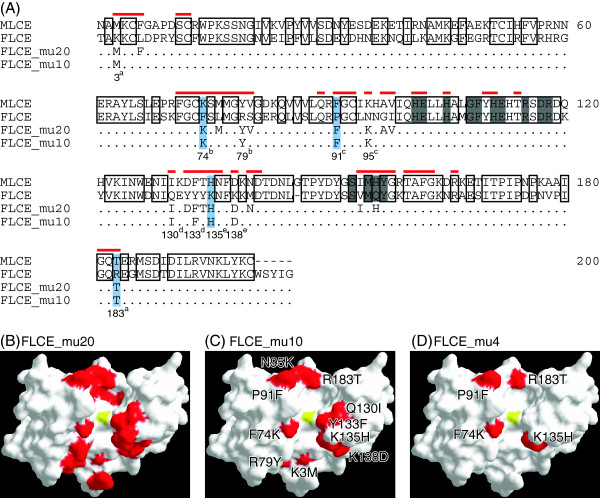
**Sequence alignment of MLCE and FLCE together with mutated sites for mutant recombinant LCEs. (A)** Sequence alignment of MLCE, FLCE, FLCE_mu20, and FLCE_mu10. Residues conserved between MLCE and FLCE are boxed with black lines. Gray boxes indicate active site consensus sequences. Red bars above the sequences indicate amino acid residues facing the active site cleft of MLCE. Amino acid residues that were mutated in FLCE_mu20 and FLCE_mu10 are also shown. The numbers and alphabetical subscripts (a–e) at the bottom of the sequences indicate two amino acid residues, which were returned to *Fundulus*-type mutations and introduced into FLCE_mu8a to mu8e. Light blue boxes indicate the mutated positions in FLCE_mu4. The amino acid residues of mutated sites in FLCE_mu20 **(B)**, FLCE_mu10 **(C)**, and FLCE_mu4 **(D)** are colored red, and the zinc atom at the catalytic center is colored yellow on the crystal structure of MLCE, shown as a surface representation.

**Figure 4:**
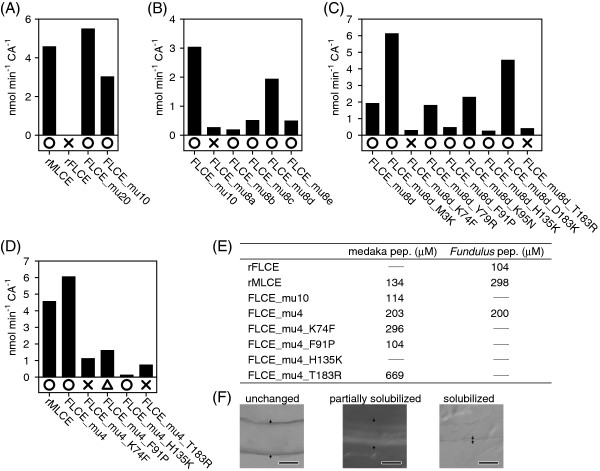
**Identification of important residues on FLCE for species-specific egg envelope digestion.** The activities of rLCEs towards the medaka mid-ZPd peptide are shown in the order of steps 1 **(A)**, 2 **(B)**, 3 **(C)**, and 4 **(D)** (see text). Circles, triangles, and crosses indicate complete, partial, and no solubilization of the swollen medaka egg envelope, respectively. **(E)** Km values (μM) of rLCEs towards medaka or *Fundulus* peptides. Bars represent “not determined”. **(F)** The morphology of unchanged, partially solubilized, or solubilized swollen envelope. Scale bars, 100 μm.

It is reasonable to hypothesize that, in addition to the P2 site of the substrate, the species-specific digestion of FLCE depends on the structure of its active site cleft, where FLCE interacts directly with the cleavage site on the egg envelope. The crystal structure of MLCE revealed that 51 residues faced the active site cleft. The residues are shown in Figure 3A. Amongst these residues, 20 substitutions were observed in MLCE or FLCE. To transform the active site cleft from *Fundulus*-type into medaka-type, we first introduced 20 amino acid mutations into FLCE, and generated recombinant mutant FLCE (rFLCE) named FLCE_mu20 (Figure 3A and 3B). FLCE_mu20 solubilized the swollen medaka egg envelope. Furthermore, the efficiency of FLCE_mu20 towards the medaka mid-ZPd peptide was not significantly different from that of recombinant MLCE (rMLCE) (Figure 4A). Therefore, we concluded that the species specificity of FLCE was caused by some of the 20 amino acid substitutions in the active site cleft.

To identify the most important mutations for species specificity, we reduced the number of mutations introduced into FLCE in a stepwise manner.

1) First, we focused on substitutions between amino acids with distinctly different properties, such as negative, positive, uncharged polar, and/or non-polar amino acids. Among the 20 substitutions, 10 sites showed such differences. These 10 residues in FLCE were substituted with medaka-type residues and named FLCE_mu10 (Figure 3A and C). FLCE_mu10 completely digested the swollen medaka egg envelope (Figure 4A). In addition, FLCE_mu10 had sufficient activity to cleave the medaka mid-ZPd peptide, and its Km value was similar to that of rMLCE (Figure 4E). Therefore, these 10 substitutions were sufficient to cause the FLCE-specific LCE activity.

2) The next strategy was as follows: two of the 10 mutations were returned to *Fundulus*-types, and named FLCE_mu10. If the returned residue(s) was an important site(s), the peptide cleavage activity of these mutant rFLCEs would decrease, and if not, the activity would not change. We generated five mutant rFLCEs, named FLCE_mu8a to 8e, whose returned sites are shown in Figure 3A. Only FLCE_mu8d did not change the peptide cleavage activity (Figure 4B). These observations suggested that some of the eight mutations (K3M, F74K, R79Y, P91F, N95K, K135H, K138D, and R183T) were important for the species specificity of FLCE.

3) Similar to the above strategy, one of the eight mutations was next returned to the *Fundulus*-type residue and named FLCE_mu8d. Eight separate mutant rFLCEs were generated, which were named FLCE_mu8d_“returned site”. As shown in Figure 4C, four of these eight mutant rFLCEs had decreased peptide cleavage activity, suggesting that F74K, P91F, K135H, and R183T were important substitutions for FLCE activity and species specificity.

4) Finally, we introduced these four mutations into FLCE and generated recombinant FLCE_mu4 (Figure 3A and B). As shown in Figure 4D, FLCE_mu4 completely solubilized the swollen medaka egg envelope, and its peptide cleavage activity was similar to that of rMLCE (Figure 4D). Therefore, the four residues at positions 74, 91, 135, and 183 were determined to be essential for the species specificity of FLCE.

To determine the contribution of the four FLCE sites to egg envelope digestion, we further individually returned one of the four sites of FLCE_mu4 to the *Fundulus* type, generating four new mutant rFLCEs, named FLCE_mu4_“returned site”. All the mutant rFLCEs decreased the peptide-cleaving activity (Figure 4D). FLCE_mu4_K74F and FLCE_mu4_T183R lost activity and, in addition, did not solubilize the swollen medaka egg envelope. The Km values of FLCE_mu4_K74F and FLCE_mu4_T183R toward the medaka peptide increased by 1.5- and 3.3-fold compared with that of FLCE_mu4, respectively, suggesting that the affinity of the mutant rFLCEs for the substrate was reduced due to the mutation at either position 74 or 183. Also, in terms of the egg envelope-solubilizing activity, residues at positions 74 and 183 appear to play a critical role in the species specificity of FLCE.

On the other hand, FLCE_mu4_F91P partially digested the swollen medaka egg envelope (Figure 4D and F), and its Km value was similar to that of rMLCE (Figure 4E). Although FLCE_mu4_H135K had the lowest activity toward the medaka mid-ZPd peptide among the four mutants, it completely solubilized the swollen medaka egg envelope. When positions 74 and 183 on rFLCE were substituted to the medaka types, mutant rFLCE (FLCE_F74K&R183T) could not solubilize the swollen medaka egg envelope. This result suggested that positions 91 and 135 are also necessary for the complete egg envelope solubilization. The cleavage of mid-ZPd may be dependent on more complicated enzyme-substrate interaction, in addition to recognition of primary sequence of the cleavage site. We conclude that these four residues are required for FLCE to maintain suitable activity towards the cleavage site peptide and to digest the swollen medaka egg envelope.

### Substrate preference of LCEs

**Figure 5:**
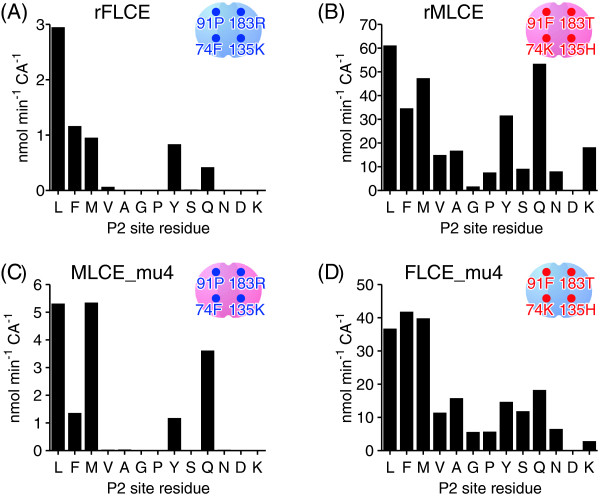
**P2 site preference of rFLCE, rMLCE, FLCE_mu4, and MLCE_mu4.** Cleavage activities of rFLCE **(A)**, rMLCE **(B)**, MLCE_mu4 **(C)**, and FLCE_mu4 **(D)** towards wild-type, shown in Figure 2C, peptide 2, and mutant medaka mid-ZPd peptides whose P2 sites were substituted with various amino acid residues. Letters under the horizontal axis show the amino acid residues at the P2 sites of the peptides. The P2 site residue of the wild-type peptide is P.

We further examined the preference of LCEs towards the P2 site using 13 medaka mid-ZPd peptide mutants. The activity of rFLCE in cleaving the peptide bond between D and S, such as EVQPXD↓SPLSI, was monitored. The minor activities of rFLCE at other cleavage sites, such as EVQPN↓DSPLSI and EVQPD↓DSPLSI, were not further investigated in the present study. rFLCE cleaved five kinds of peptides, whose P2 site residues were L, F, M, Y, or Q (Figure 5A). On the other hand, rMLCE cleaved the majority of the peptides, and its activity was higher than that towards wild-type mid-ZPd peptide with P at the P2 site (Figure 5B). Therefore, we determined that rMLCE had a broader P2 site preference than rFLCE. rFLCE preferentially cleaved the peptides with large hydrophobic residues at the P2 site. Another characteristic difference was in the efficiency of the peptide cleavage. The activity of MLCE was about 10 times greater than that of FLCE. Thus, MLCE and FLCE are different not only in substrate preference, but also in peptide cleavage efficiency.

Next, we evaluated the P2 site preference of LCE by introducing four mutations at positions 74, 91, 135, and 183 that are important for the species specificity of LCE. When medaka-type residues were introduced into *Fundulus* FLCE (FLCE_mu4), its activity was similar to that of medaka rMLCE (Figure 5D). When *Fundulus*-type residues were introduced into medaka MLCE (MLCE_mu4), its activity was altered to that of the *Fundulus* type (Figure 5C). Therefore, we confirmed that the P2 site preference of LCEs was determined by the four amino acid residues in LCE.

### A co-evolutionary pathway for hatching enzyme and egg envelope protein

**Figure 6:**
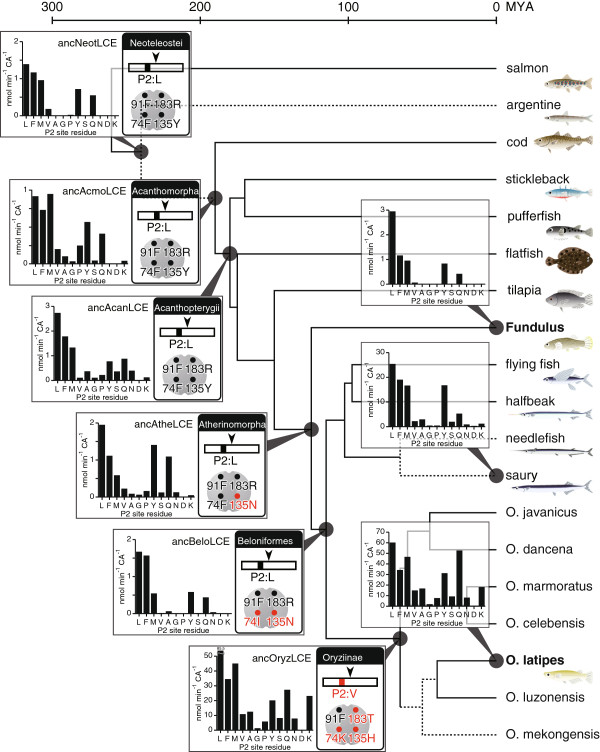
**Predicted evolutionary pathway for hatching enzyme and egg envelope protein in the euteleostean lineage.** The phylogram of euteleosts was simplified from the mitogenomic phylogeny of fishes [30,31]. Divergence time was according to Setiamarga et al. [30], and the lineages drawn with dotted lines are hypothetical. Amino acid residues of the ancestral P2 site and those of positions 74, 91, 135, and 183 on ancestral LCEs are shown in the boxes at the nodes. The residues for the ancestor of neoteleosts are colored black, and the residues substituted during evolution are colored red. P2 site preferences of ancestral LCEs of Neoteleostei (ancNeotLCE), Atherinomorpha (ancAtheLCE), Beloniformes (ancBeloLCE), and Oryziinae (ancOryzLCE) are shown at each node.

Both genes for hatching enzymes and egg envelope proteins were cloned from various euteleostean species (Additional file 1: Figure S1). We deduced the ancestral sequences using PAML. Figure 6 illustrates the order of changes in amino acids introducing species-specific egg envelope digestion during the evolution of teleostean fishes. The P2 site of the mid-ZPd site in egg envelope protein was predicted to have been occupied by L in the ancestral euteleostean species. These residues were well conserved in most of the euteleostean sequences investigated in this study. The only observed difference occurred during the evolution into Oryziinae, where there was a substitution to V or P.

The ancestral euteleostean LCE was predicted to have had 74 F, 91 F, 135Y, and 183R in its active site cleft. These essential residues were well conserved prior to Atherinomorpha. The ancestor of Atherinomorpha was characterized by a single substitution at position 135. Later, another substitution occurred at position 74 in the ancestor of Beloniformes. Finally, three further substitutions occurred in the ancestor of Oryziinae. These observations suggested that substitutions of the four essential residues in hatching enzyme LCE occurred prior to substitutions in the cleavage site on egg envelope protein.

### Activity of ancestral recombinant LCE

Finally, we generated recombinant ancestral LCEs based on the predicted ancestral sequences (Additional file 2: Figure S2) and examined their P2 site preference (Figure 6). The ancestral LCE of Neoteleostei (ancNeotLCE) had strong P2 site preference, similar to that of FLCE. However, the P2 site preference of ancestral LCE of Acanthomorpha (ancAcmoLCE) was less stringent, and the ancestral LCE of Acanthopterygii (ancAcanLCE) had cleavage activity towards various substrates. Again, in the ancestor of Beloniformes (ancBeloLCE), high P2 site preference was revived, but was dramatically altered in the ancestor of Oryziinae, resulting in broader substrate preference. In summary, the MLCE-like activity had been established in the ancestor of Oryziinae, and this ancestral LCE (ancOryzLCE) acquired the ability to cleave the peptide containing P at the P2 site. Therefore, it is reasonable to conclude that the acquisition of this new ability would permit the substitution to P at the P2 site of the ancestor of the medaka egg envelope protein.

## Discussion

The present work demonstrated the molecular basis of species-specific egg envelope digestion by *Fundulus* hatching enzyme FLCE toward medaka egg envelope. The species specificity of FLCE depended on both the P2 site in the LCE-cleavage site on the egg envelope protein, and four sites in the active site cleft of LCE (positions 74, 91, 135, and 183). Generation of mutant recombinant enzymes suggested that positions 74 and 183 were indispensable for LCE activity. We further compared these residues among euteleosts (Additional file 1: Figure S1C). In most euteleosts, the residues at position 74 were hydrophobic; only medaka LCE contained a basic residue at this position. The residue at position 183 was well conserved, and was found to be R in most teleosts. However, the residue was occupied by S or T in *beloniform* LCEs, including medaka. The residue at position 91, which is the third important site for species specificity, has been substituted to P in *Fundulus* LCE (Additional file 1: Figure S1C). The site with the lowest contribution to specificity (position 135) was substituted to a basic residue in *Fundulus* (K) and medaka (H) (Additional file 1: Figure S1C).

We next examined how these substitutions were introduced into LCE during evolution, changing its specificity. We analyzed ancestral LCEs by reconstructing the proteins and examining their activities. As shown in Figure 6, the activity of the ancestral LCE of Beloniformes (ancBeloLCE) had a high P2 site preference. In the ancestor of Oryziinae, three substitutions (74 K, 135H, and 183 T) occurred in LCE, introducing a lower P2 site preference and high specific activity into LCE. The substitution from F (codon TTY) to K (codon AAR) at position 74 required a two-step nucleotide substitution. This two-step process was predicted by PAML to be a substitution from F (TTC) to I (ATC), and then to K (AAG). Such substitutions in LCE tolerated the substitution of V into the LCE cleavage site of the egg envelope, and then the substitution to P occurred during the evolution of Oryziinae.

We compared the species specificity of LCE in egg envelope digestion among several pairs of fishes. Three pairs (three-spined stickleback vs. medaka *O. latipes*, three-spined stickleback vs. nine-spined stickleback, and medaka *O. latipes* vs. saury) showed species-specificity, while two (*O. latipes* vs. *O. javanicus*, and *Fundulus* vs. saury) did not. The results suggested that the pairs within genus *Oryzias* and those within other euteleosts showed no species specificity, except the pair between three- and nine-spined sticklebacks, while those between *Oryzias* species and other euteleosts showed species specificity. Therefore, the manner of the establishment of the LCE species specificity found in *Fundulus* and medaka seems to be applicable to the other fish pairs, except the three- and nine-spined stickleback pair. As we only examined a few pairs of fishes, we cannot rule out the possibility that the LCE species specificity arose independently in different pairs of fishes in each co-evolutionary pathway.

**Figure 7:**
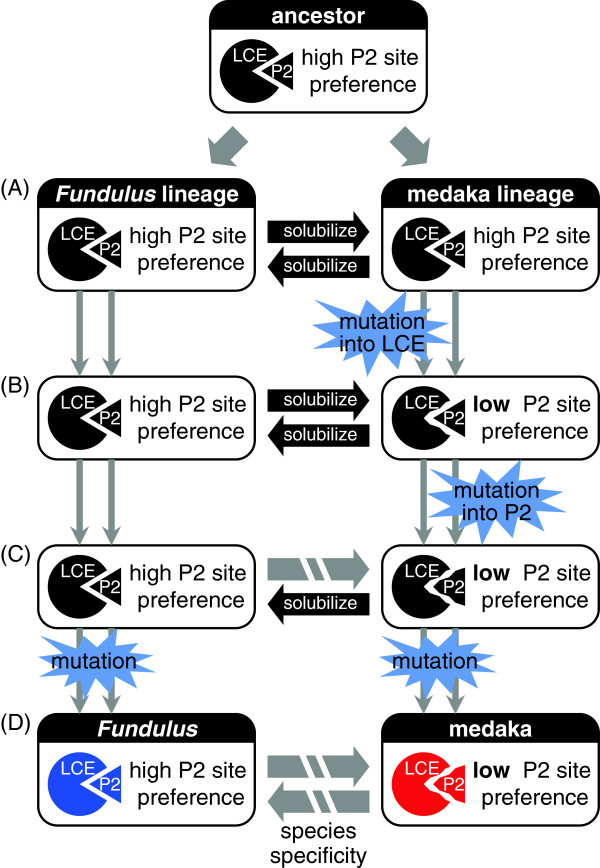
**Model of co-evolution of hatching enzyme and egg envelope protein. (A)** Ancestral hatching enzyme (LCE) and egg envelope protein (P2) co-evolved in both *Fundulus* and medaka lineages. **(B)** In the medaka lineage, mutation in LCE introduced lower P2 site preference while maintaining the activity of LCE in solubilizing the egg envelope. **(C)** Then, mutation in egg envelope protein occurred in the medaka lineage, and ancestral *Fundulus* LCE became unable to digest ancestral medaka egg envelope. **(D)** In both *Fundulus* and medaka lineages further substitutions occurred, and strict species specificity was established between the two species.

Finally, we illustrated how amino acid substitutions introduced species specificity into LCE of *Fundulus* and medaka during evolution. As summarized in Figure 7, a co-evolutionary process to establish species-specific egg envelope digestion was initiated by substitution in LCE (Figure 7A and 7B). Substitutions occurring in the pathway to Oryziinae lowered the P2 site preference and, later, allowed further substitutions in the substrate. Indeed, such substitutions in the substrate occurred during the evolution into Oryziinae (Figure 7B and 7C).

The substitution in the substrate established the species specificity of FLCE. FLCE maintained the ancestral activity with high substrate specificity and was unable to digest medaka egg envelope (Figure 7C). Further substitutions in the enzyme and/or substrate conferred higher specificity on the interaction between enzyme and substrate, and thus MLCE could not digest *Fundulus* envelope (Figure 7C and 7D). In such a manner, the strict species specificity was established in *Fundulus* and medaka (Figure 7D). Determination of the substitution sites responsible for species specificity of medaka enzyme is in progress.

It is generally accepted that strategies such as mutation and subsequent production of a recombinant mutant protein, followed by the evaluation of its function, are required to understand the evolution of protein function at the molecular level [23]. At present, such strategies have been extended to studies on reconstructed probable ancestors of the protein. One example is a study on hormone-receptor complexes, in which glucocorticoid and mineralocorticoid receptors switched their ligand preference following two amino acid substitutions [24]. Another study showed that several sites are important for recognition of sugar by conger eel galectins [23]. The former study suggested that several amino acid substitutions are required for switching the ligand preference or specificity, possibly accompanied with some conformational change of the 3D structure of the protein itself [24]. In the present study we demonstrated that, in the hatching enzyme LCE-egg envelope protein pair, four substitutions in the LCEs allowed one amino acid substitution at the LCE-cleavage site on the substrate, egg envelope, during evolution. Interestingly, all four substitutions were located at the edge of the active site cleft, but not within the active site cleft itself, where some amino acid side chains might interact directly with those of the substrate around the cleavage site. These substitutions are likely to result in minor but not drastic changes in substrate specificity, suggesting that the enzyme activities have gradually been changed during evolution. A similar study reported that the substitutions responsible for environmental adaptation of fish lactate dehydrogenase are located outside of the active site cleft, and induced structural changes of the active site cleft [25].

In experiments to reconstruct ancestral LCE, ancNeotLCE showed strong P2 site preference. During evolution, the P2 site preference became less stringent in ancAcmoLCE, and then stronger again in ancBeloLCE, then lower in ancOryzLCE, i.e., P2 site preference seems to have fluctuated. These results imply that the enzyme has been continually changing during evolution while still maintaining its function in cleavage of the mid-ZPd site. When the substrate mutation fit with a change of enzyme specificity, the substrate mutation would be fixed, and then a new substrate-enzyme pair would be generated during later co-evolution. Therefore, the co-evolution of proteins is dependent on the evolution of protein-protein interaction, although this can sometimes be very complicated. The present study provides a molecular basis for understanding the mechanisms involved in protein-protein co-evolution.

## Conclusions

The present study provides a molecular mechanism of co-evolution of a protease and its substrate using the interaction of hatching enzyme with egg envelope protein as a model. Four substitutions in LCE and a single substitution within egg envelope protein observed in medaka and *Fundulus* were responsible for the species-specific egg envelope digestion. We further observed how these residues were changed during the evolution of teleosts, and suggested that substitutions of the four essential residues on LCE occurred prior to substitutions in the cleavage site of egg envelope protein. The activities of predicted ancestral recombinant proteins were consistent with these proposed changes. Finally, we proposed a model for the protease-substrate co-evolution, whereby the evolutionary process leading to the species-specific action of LCE was initiated by amino acid substitutions in LCE, resulting in altered substrate specificity, which later allowed an amino acid substitution in the LCE-cleavage site of the egg envelope.

## Methods

### Fishes

*Fundulus heteroclitus* embryos were supplied by the National Research Institute of Fisheries Science, Fisheries Research Agency, Japan. Saury (*Cololabis saira*) embryos and adults were obtained from Miyako Station, National Center for Stock Enhancement, Fisheries Research Agency, Japan. *Oryzias* species were supplied by the National BioResource Project (medaka) in Japan. *O. luzonensis*, *O. mekongenesis*, *O. celebensis*, and *O. marmoratus* were from Niigata University, while *O. javanicus* were from the National Institute for Basic Biology. Tissue samples of flying fish (*Cypselurus agoo*), halfbeak (*Hyporhamphus sajori*), and needlefish (*Ablennes hians*) were obtained from the National Museum of Nature and Science, Japan. Genomic DNA sequence from argentines (*Glossanodon semifasciatus*) was derived from Ishiguro et al., 2003 [26].

### Estimation of egg envelope digestion activity of hatching enzymes

Hatching enzymes from medaka (MHCE and MLCE) and *Fundulus* (FHCE1/2 and FLCE) were purified from hatching liquids (culture medium of embryos immediately after hatching) according to a method described previously [13,14,21]. The egg envelope digestion activity of HCE was determined by turbidimetry, using fragments of fertilized egg envelope as substrate [27]. The specific activity was expressed as ΔT_610_ · μg protein^-1^. For LCE activity, the amounts of peptides liberated from the swollen envelope were measured [27], and the specific activity was expressed as ΔA_595_ · μg protein^-1^.

### Caseinolytic activity

The caseinolytic activity (CA) of hatching enzyme was measured in a 750 μL reaction mixture consisting of 83 mM Tris–HCl (pH 8.0) and 3.3 mg mL^-1^ of casein, according to a method described previously [21]. CA was expressed in terms of ΔA_280_ · 30 min^-1^.

### Synthetic peptide-cleaving activity

A 40-μL reaction mixture containing 100 μM of synthetic peptide in 50 mM Tris–HCl (pH 8) and an appropriate amount of enzyme was used to measure the activity according to a method described previously [21]. To determine the Michaelis constant (Km), synthetic peptides were used as substrates in a concentration range from 10–1000 μM. Consumption of substrate was limited to 30 % of the starting concentration. The Km values were determined by Lineweaver-Burk plot.

### Recombinant protein

The native nucleotide sequences of FLCE and MLCE were partially optimized for *Escherichia coli* codon preferences, without changing the deduced amino acid sequence. The mature enzyme region, containing suitable restriction enzyme sites (*Bam*HI and *Nd*eI) at both ends of the cDNA, was cloned into pET3c for expression in *E. coli*. Mutant FLCE or MLCE plasmids were constructed by PCR using mutagenic primers and codon-optimized FLCE or MLCE plasmids as a template. The recombinant proteins were synthesized using a protocol described previously [27]. Because the refolding efficiencies of mutant recombinant proteins differed from sample to sample, the amount of active enzyme in the sample was estimated by CA. To compare the peptide-cleaving efficiency of the mutant recombinant proteins, the activities were normalized by CA, and expressed as nmol min^-1^ CA^-1^.

### Cloning of hatching enzyme and choriogenin genes

Hatching enzyme cDNAs from saury, *O. javanicus*, and *O. dancena* were cloned by rapid amplification of cDNA ends (RACE) PCR, followed by RT-PCR, from RNA of pre-hatching embryos, according to a previously described method [28]. LCE gene fragments from flying fish, needlefish, *O. luzonensis*, *O. mekongenesis*, *O. celebensis*, and *O. marmoratus* were amplified by PCR from genomic DNA using primers designed from MLCE. Gene fragments for the egg envelope precursor choriogenin H (ChgH) were amplified from genomic DNA of argentines, saury, flying fish, halfbeak, needlefish, *O. luzonensis*, *O. mekongenesis*, *O. celebensis*, and *O. marmoratus* using primers designed from conserved regions in ChgH genes, as described previously [21].

### Ancestral sequence inference

Ancestral nucleotide sequences of LCE and ChgH were inferred by maximum likelihood using PAML 4.4 software [29], based on the molecular phylogeny of 33 euteleostean fish species for LCE, and 30 species for ChgH, according to Setiamarga et al. [30] and Takehana et al. [31]. For ancestral reconstruction, the GTR model was assumed. Amino acid sequences were deduced from the inferred ancestral nucleotide sequences.

## Abbreviations

HCE: High choriolytic enzyme; LCE: Low choriolytic enzyme; MHCE: Medaka high choriolytic enzyme; MLCE: Medaka low choriolytic enzyme; FHCE: *Fundulus* high choriolytic enzyme; FLCE: *Fundulus* low choriolytic enzyme; CA: Caseinolytic activity; ZP: Zona pellucida; ChgH: Choriogenin H.

## Competing interests

The authors declare that they have no competing interests.

## Authors’ contributions

MK, II, MN, and SY participated in the design of the experiments. MK and KI cloned the genes. MK performed biochemical experiments. MK and SY analyzed the data. MK, II, MN, and SY wrote the paper. All authors read and approved the final manuscript.

## Supplementary Material

Additional file 1: Figure S1 Alignment of partial amino acid sequences around mid-ZPd with that of LCE. Partial amino acid sequences around mid-ZPd in ZPB are compared among euteleosts **(A)** and Oryziinae species **(B)**. The LCE cleavage site is shown as an arrowhead, and the P2 site is indicated by a gray box. Partial amino acid sequences of LCE were compared among euteleosts **(C)** and Oryziinae species **(D)**. Positions 74, 91, 135, and 183 are indicated by gray boxes. Identical residues are boxed. O.mykiss, *Oncorhynchus mykiss*; O.masou, *Oncorhynchus masou*; Salmo, *Salmo salar*; Esox, *Esox americanus*; Plecoglossus, *Plecoglossus altivelis*; Spirinchus, *Spirinchus lanceolatus*; Hypomesus, *Hypomesus nipponensis*; Glossanodon, *Glossanodon semifasciatus*; Gadus, *Gadus macrocephalus*; Liparis, *Liparis atlanticus*; Helicolenus, *Helicolenus hilgendorfi*; Setarches, *Setarches guentheri*; Gasterosteus, *Gasterosteus aculeatus*; P.sinensis, *Pungitius sinensis*; P.pungitius, *Pungitius pungitius*; Culaea, *Culaea inconstans*; Spinachia, *Spinachia spinachia*; Apeltes, *Apeltes quadracus*; Sparus, *Sparus aurata*; Tetraodon, *Tetraodon nigroviridis*; Takifugu, *Takifugu rubripes*; Verasper, *Verasper variegatus*; Paralichthys, *Paralichthys olivaceus*; Pseudopleuro, *Pseudopleuronectus americanus*; Oxyeleotris, *Oxyeleotris marmoratus*; Oreochromis, *Oreochromis niloticus*; Fundulus, *Fundulus heteroclitus*; Cypselurus, *Cypselurus agoo*; Hyporhamphus, *Hyporhamphus sajori*; Ablennes, *Ablennes hians*; Cololabis, *Cololabis saira*; O.latipes, *Oryzias latipes*; O.luzonensis, *Oryzias luzonensis*; O.mekongensis, *Oryzias mekongenesis*; O.celebensis, *Oryzias celebensis*; O.marmoratus, *Oryzias marmoratus*; O.javanicus, *Oryzias javanicus*; O.dancena, *Oryzias dancena*. Click here for file

Additional file 2: Figure S2 Alignment of mature enzyme regions of ancestral LCEs. Amino acid sequences of mature enzyme regions of predicted ancestral LCE were compared among the ancestors of Euteleostei (ancEuteLCE), Neoteleostei (ancNeotLCE), Atherinomorpha (ancAtheLCE), Beloniformes (ancBeloLCE), and Oryziinae (ancOryzLCE). Positions 74, 91, 135, and 183 are indicated by gray boxes. Identical residues are boxed. Click here for file
