# Supplementary material for: Molecular co-evolution of a protease and its substrate elucidated by analysis of the activity of predicted ancestral hatching enzyme
Source: BMC Evol Biol. 2013 Oct 25;13:231. doi: 10.1186/1471-2148-13-231 (PMC3819744; doi:10.1186/1471-2148-13-231)

Fig. S1 Kawaguchi et al

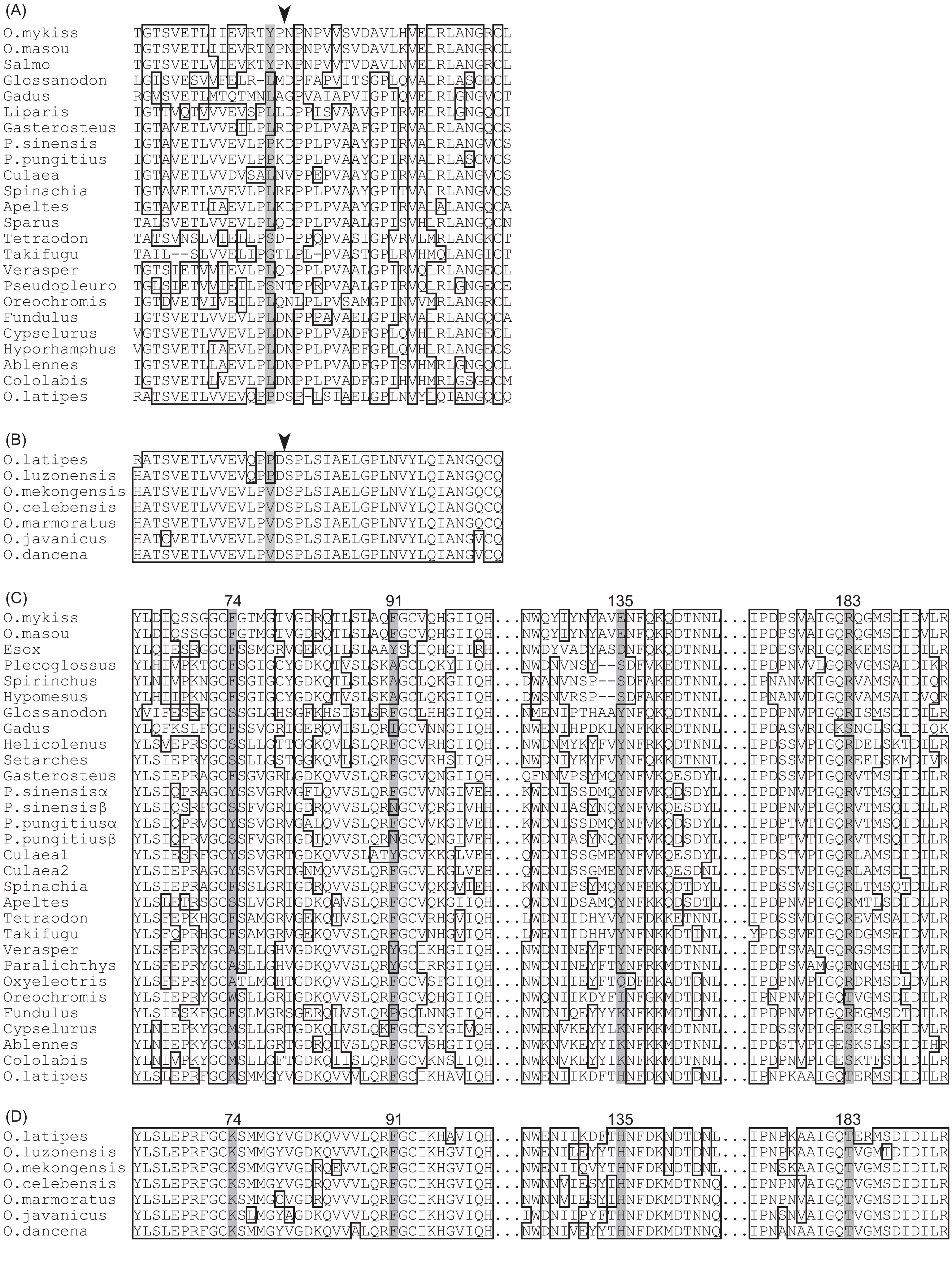

Supplement: Additional file 1: Figure S1 — Alignment of partial amino acid sequences around mid-ZPd with that of LCE. Partial amino acid sequences around mid-ZPd in ZPB are compared among euteleosts (A) and Oryziinae species (B). The LCE cleavage site is shown as an arrowhead, and the P2 site is indicated by a gray box. Partial amino acid sequences of LCE were compared among euteleosts (C) and Oryziinae species (D). Positions 74, 91, 135, and 183 are indicated by gray boxes. Identical residues are boxed. O.mykiss, Oncorhynchus mykiss; O.masou, Oncorhynchus masou; Salmo, Salmo salar; Esox, Esox americanus; Plecoglossus, Plecoglossus altivelis; Spirinchus, Spirinchus lanceolatus; Hypomesus, Hypomesus nipponensis; Glossanodon, Glossanodon semifasciatus; Gadus, Gadus macrocephalus; Liparis, Liparis atlanticus; Helicolenus, Helicolenus hilgendorfi; Setarches, Setarches guentheri; Gasterosteus, Gasterosteus aculeatus; P.sinensis, Pungitius sinensis; P.pungitius, Pungitius pungitius; Culaea, Culaea inconstans; Spinachia, Spinachia spinachia; Apeltes, Apeltes quadracus; Sparus, Sparus aurata; Tetraodon, Tetraodon nigroviridis; Takifugu, Takifugu rubripes; Verasper, Verasper variegatus; Paralichthys, Paralichthys olivaceus; Pseudopleuro, Pseudopleuronectus americanus; Oxyeleotris, Oxyeleotris marmoratus; Oreochromis, Oreochromis niloticus; Fundulus, Fundulus heteroclitus; Cypselurus, Cypselurus agoo; Hyporhamphus, Hyporhamphus sajori; Ablennes, Ablennes hians; Cololabis, Cololabis saira; O.latipes, Oryzias latipes; O.luzonensis, Oryzias luzonensis; O.mekongensis, Oryzias mekongenesis; O.celebensis, Oryzias celebensis; O.marmoratus, Oryzias marmoratus; O.javanicus, Oryzias javanicus; O.dancena, Oryzias dancena. [file 1471-2148-13-231-S1.pdf]
