# Supplementary material for: Molecular co-evolution of a protease and its substrate elucidated by analysis of the activity of predicted ancestral hatching enzyme
Source: BMC Evol Biol. 2013 Oct 25;13:231. doi: 10.1186/1471-2148-13-231 (PMC3819744; doi:10.1186/1471-2148-13-231)
Supplement: Additional file 2: Figure S2 — Alignment of mature enzyme regions of ancestral LCEs. Amino acid sequences of mature enzyme regions of predicted ancestral LCE were compared among the ancestors of Euteleostei (ancEuteLCE), Neoteleostei (ancNeotLCE), Atherinomorpha (ancAtheLCE), Beloniformes (ancBeloLCE), and Oryziinae (ancOryzLCE). Positions 74, 91, 135, and 183 are indicated by gray boxes. Identical residues are boxed. [file 1471-2148-13-231-S2.pdf]

Fig. S2 Kawaguchi et al

```
ancEuteLCE  NAMKCFRNKYSCLWKKSANGMVDVPYIISDVYSSVEEETIETAMKDFHCKTCIRFIPRKT
ancNeotLCE  NAMKCFRKKYSCLWKKSANGKVEVPYIISDVYSSAEKETIKNAMKDFHSKTCIRFVPRQT
ancAcmoLCE  NAMKCFSKTYSCLWPKSANGKVEIPFIISEKYADTEKKAILNAMKDFDSKTCIRFVPRKT
ancAcanLCE  NAMKCFNKTYSCLWPKSANGNVEIPFIISEKYDDTEKNAILTAMKDFECKTCIRFVPRKT
ancAtheLCE  NAMKCFNKPYSCLWPKSSNGNVEIPFIISEKYDDTEKNTILNAMKGFECKTCIRFVPRRT
ancBeloLCE  NAMKCFNKPYSCLWPKSSNGNVEIPFIISEKYDDTEKNTILNAMKGFESKTCIRFVPRRT
ancOryzLCE  NAMKCFGDPDSCRWPKSSNGIVKVPYVVSDNYDNSEKETIRNAMKEFAEKTCIHFVPRNN

                         74               91
ancEuteLCE  QRAYLNIESRGGCFSSMGRVGDKQTLSLARFGCVQHGIIQHELLHALGFYHEHTRSDRDQ
ancNeotLCE  QRAYLNIESRYGCFSGMGRIGDKQTVSLSRFGCVQHGIIQHELLHALGFYHEHTRSDRDQ
ancAcmoLCE  QRAYLNIEPRYGCFSLMGRIGDKQVVSLQRFGCVQHGIIQHELLHALGFYHEHTRSDRDQ
ancAcanLCE  QRAYLSIEPRYGCFSLLGRIGDKQVVSLQRFGCVRHGIIQHELLHALGFYHEHTRSDRDQ
ancAtheLCE  ERAYLSIEPRYGCFSLLGRTGDKQVVSLQRFGCVNHGIIQHELLHALGFYHEHTRSDRDQ
ancBeloLCE  ERAYLSIEPRYGCISLLGRTGDKQVVSLQRFGCVNHGIIQHELLHALGFYHEHTRSDRDQ
ancOryzLCE  ERAYLSLEPRFGCKSMMGYVGDKQVVVLQRFGCIKHGVIQHELLHALGFYHEHTRSDRDQ

                         135
ancEuteLCE  YVRINWENIYNYAVYNFQKQDTNNLNTPYDYSSVMHYGRTAFTNYHRKETITPIPDPSVP
ancNeotLCE  YVRINWENIHNYAVYNFQKQDTNNLNTPYDYSSVMHYGRTAFTNYRRKETITPIPDPSVP
ancAcmoLCE  YVRINWENINDYFVYNFQKKDTNNLNTPYDYSSVMHYGRTAFGKYGRKETITPIPDPSVP
ancAcanLCE  YVRINWENINEYFVYNFQKKDTNNLNTPYDYSSVMHYGRTAFGKYGRTETITPIPDPSVP
ancAtheLCE  YVRINWENIKEYYINNFKKMDTNNLDTPYDYSSVMHYGRTAFGKYGRTETITPIPDPTVP
ancBeloLCE  YVRINWENIKEYYINNFKKMDTNNLDTPYDYSSVMHYGRTAFGKYGRTETITPIPDPTVP
ancOryzLCE  HVKINWENIIEYYTHNFDKMDTNNLGTPYDYGSIMHYGRTAFGK-DRKETITPIPNPNVA

              183
ancEuteLCE  IGQRKGMSDIDILRINKLYKC
ancNeotLCE  IGQREGMSDIDILRINRLYKC
ancAcmoLCE  IGQREGMSDIDILRINRLYKC
ancAcanLCE  IGQREGMSDIDILRINRLYKC
ancAtheLCE  IGQREGMSDIDILRINRLYKC
ancBeloLCE  IGQREGMSDIDILRINRLYKC
ancOryzLCE  IGQTVGMSDIDILRVNKLYKC
```
